# Hypothermia-Induced Ubiquitination of Voltage-Dependent Anion Channel 3 Protects BV2 Microglia Cells From Cytotoxicity Following Oxygen-Glucose Deprivation/Recovery

**DOI:** 10.3389/fnmol.2020.00100

**Published:** 2020-06-05

**Authors:** Shen Zhao, Peng Xiao, Hao Cui, Ping Gong, Caijing Lin, Feng Chen, Ziren Tang

**Affiliations:** ^1^Shengli Clinical Medical College, Fujian Medical University, Fuzhou, China; ^2^Department of Emergency Medicine, Fujian Provincial Hospital, Fujian Institute of Emergency Research, Fuzhou, China; ^3^Department of Emergency Medicine, Beijing Chao-Yang Hospital, Capital Medical University, Beijing, China; ^4^Beijing Key Laboratory of Cardiopulmonary Cerebral Resuscitation, Beijing Chao-Yang Hospital, Capital Medical University, Beijing, China; ^5^Department of Emergency Medicine, the First Affiliated Hospital of Dalian Medical University, Dalian City, China

**Keywords:** hypothermia, VDAC3, BV2 microglial cells, oxygen-glucose deprivation/recovery, cytotoxicity

## Abstract

**Background**: Hypothermia attenuates microglial activation and exerts a potential neuroprotective effect against cerebral ischemic-reperfusion (I/R) injury. However, the underlying mechanism remains to be elucidated. In this *in vitro* study, a model of oxygen-glucose deprivation, followed by recovery (OGD/R), was used to investigate whether hypothermia exerts anti-inflammatory and anti-apoptosis properties *via* enhanced ubiquitination and down-regulation of voltage-dependent anion channel 3 (VDAC3) expression.

**Methods**: BV2 microglia were cultured under OGD for 4 h following reperfusion with or without hypothermia for 2, 4, or 8 h. M1 and M2 microglia markers [inducible nitric oxide synthase (iNOS) and arginase (Arg)1] were detected using immunofluorescence. The levels of pro-inflammatory cytokines [tumor necrosis factor (TNF) α, interleukin (IL)-1β], and anti-inflammatory factor (IL-10) were determined using enzyme-linked immunosorbent assay (ELISA). Mitochondrial membrane potential (ΔΨm) was assayed by JC-1 staining using a flow cytometer. Expression of caspase-3, cleaved caspase-3, and VDAC3 were assessed using western blot analysis. The cellular locations and interactions of ubiquitin and VDAC3 were identified using double immunofluorescence staining and immunoprecipitation (IP) assay. Also, the level of the VDAC3 mRNA was determined using a quantitative polymerase chain reaction (qPCR).

**Results**: Hypothermia inhibited the OGD/R–induced microglia activation and differentiation into the M1 type with pro-inflammatory effect, whereas it promoted differentiation to the M2 type with anti-inflammatory effect. Hypothermia attenuated OGD/R-induced loss of Δψm, as well as the expression of apoptosis-associated proteins. Compared to normothermia, hypothermia increased the level of ubiquitinated VDAC3 in the BV2 microglia at both 2 and 8 h of reperfusion. Furthermore, hypothermia did not attenuate VDAC3 mRNA expression in OGD/R-induced microglia.

**Conclusions**: Hypothermia treatment during reperfusion, attenuated OGD/R-induced inflammation, and apoptosis in BV2 microglia. This might be due to the promotion of VDAC3 ubiquitination, identifying VDAC3 as a new target of hypothermia.

## Introduction

Neurological injury resulting from the successful return of spontaneous circulation (ROSC) after cardiac arrest (CA) remains a leading cause of long-term disability and death worldwide (Keijzer et al., 2018; Sandroni et al., 2018). With the rapid development of imaging methods such as positron emission tomography (PET) and two-photon microscopy, it is practicable to study microglia activation during experimental and clinical ischemia-reperfusion (I/R; Wake et al., 2009; Thiel et al., 2010). Following reperfusion, the activated microglia participates in infiltrated inflammation, oxidative stress, and apoptosis, inducing brain damage (Surinkaew et al., 2018). Furthermore, microglial activation and brain damage were aggravated under both hyperoxic resuscitation and hyperthermia (Hazelton et al., 2010; Kim et al., 2015).

Therapeutic hypothermia (TH) for comatose patients following ROSC after CA has the potential to reduce neurological morbidity and mortality (Callaway et al., 2015). However, recent TH clinical trials presented neutral results at different durations of hypothermia (Kirkegaard et al., 2017). Furthermore, the effect of different durations of hypothermia on microglial activation and the neurological outcome is undefined. Thus, further studies are required to understand the implicit mechanism of TH for treating cerebral I/R.

The voltage-dependent anion channel (VDAC) family is the most prevalent ion channel in the outer mitochondrial membrane (OMM). The three VDAC isoforms (VDAC1, 2, and 3) share high structural homology in mammals (Bayrhuber et al., 2008; Neumann et al., 2010). VDAC can modulate the mitochondrial membrane permeability (MMP), playing a role in the conduction of metabolites and maintenance of homeostasis. Thus, VDAC can be considered a potential target for the onset of ischemia/reperfusion injury, trauma, and neurodegenerative diseases (Uchino et al., 2008; Karachitos et al., 2017). The VDAC family has also been identified in microglial cells (Rimmerman et al., 2013). A recent study showed that activated VDAC was temperature-sensitive, which might contribute to hypothermic neuroprotection under various pathological conditions (Imada et al., 2010; Sato-Numata et al., 2014).

Our previous study showed that hypothermia inhibits ischemia-induced increases in MMP, which provided neuroprotection against cerebral injury in a swine model of cardiac arrest (Gong et al., 2013). In this study, we hypothesized that VDAC3 in microglia may influence cell activation and neuronal injury from oxygen-glucose deprivation/recovery (OGD/R). Hence, we aimed to investigate the underlying mechanism using different durations of TH for treating cerebral I/R.

## Materials and Methods

### Materials

The materials used in this study are as follows: BV2 microglial cell lines (Procell, Wuhan, China); high glucose Dulbecco’s modified Eagle’s medium (DMEM; Hyclone, Logan, UT, USA); glucose-free DMEM (Jinuo Technologies, Hangzhou, China); 10% fetal bovine serum (FBS; Gibco Grand Island, NY, USA); cell counting kit-8 (CCK-8, Dojindo Molecular Technologies, Tokyo, Japan); 5,5′,6,6′-tetrachloro-1,1′,3,3′-tetraethyl-benzimidazolylcarbocyanine iodide (JC-1, Beyontime, Shanghai, China); tumor necrosis factor (TNF) α, interleukin (IL)-1β and (IL)-10 enzyme-linked immunosorbent assay (ELISA) kits (R & D Systems, Minneapolis, MN, USA); rabbit anti-induced nitric oxide synthase (iNOS) antibody (Proteintech, Wuhan, China); rabbit anti-arginase 1 (Arg1) antibody (Proteintech, Wuhan, China); mouse anti-ubiquitin antibody (Thermo Fisher Scientific, Waltham, MA, USA); rabbit anti-VDAC3 antibody (Aviva Systems Biology, San Diego, CA, USA); rabbit anti-caspase3 antibody (Proteintech, Wuhan, China); mouse anti-beta actin antibody (Abcam, Cambridge, UK); CoraLite594-conjugated goat anti-rabbit (red) IgG (Proteintech, Wuhan, China); CoraLite488-conjugated goat anti-mouse (green) IgG (Proteintech, Wuhan, China); horseradish peroxidase-conjugated goat anti-rabbit or anti-mouse IgG (Abcam, Cambridge, UK); SureBeads Protein G Magnetic Beads (Bio-Rad Laboratories, Hercules, CA, USA); RNAprep Pure Cell kit (Tiangen Biotech, Beijing, China); first strand cDNA synthesis kit (Abm, Richmond, BC, Canada); EvaGreen 2× quantitative polymerase chain reaction (qPCR) master mix kit (Abm, Richmond, BC, Canada).

### Cell Culture and Passage

The murine BV2 microglial cells were cultured in T-25 tissue culture cell flasks using high glucose DMEM supplemented with 10% FBS and cultured at 37°C in a 5% CO_2_ incubator (data see the Supplementary Table S1). The microglial cells were maintained *via* 2–3 passages each week. Cells from passages 3–6 were collected for subsequent experiments. Cells were seeded into 96-well plates (5 × 10^3^ cells/well for CCK-8 assay), 24-well plates (1 × 10^4^ cells/well for immunofluorescence, 5 × 10^5^ cells/well for ELISA and qPCR), 6-well plates (2 × 10^6^ cells/well for flow cytometry), or 100-mm culture dishes [1 × 10^7^ cells/well for immunoprecipitation (IP) and immunoblotting]. The cell count, images capture, and quantitative analyses were performed *via* a double-blinded approach. Each experiment was repeated a total of five times. For cell viability assay, six wells were used for each group; for other assays, three wells were used for each group.

### OGD/R and Experimental Grouping

For the OGD/R experiments, the cells were incubated in serum- and glucose-free DMEM pre-gassed with 95% N_2_ and 5% CO_2_ for ≥5 min, and then placed in a hypoxia chamber (BioSpherix C21, USA) filled with 5% CO_2_ and 0.3% O_2_ at 37°C for 4 h. Next, the cells were returned to the incubator (74% N_2_/21% O_2_/5% CO_2_) with normal cell culture medium at 37°C (normothermia, NT) or 34°C (hypothermia, HT) and incubated for 2, 4, or 8 h. Accordingly, BV2 microglial cells were divided into eight groups: Sham, OGD, OGD/R2h-NT, OGD/R4h-NT, OGD/R8h-NT, OGD/R2h-HT, OGD/R4h-HT, and OGD/R8h-HT. The OGD cells only received serum-glucose deprivation plus hypoxia, and the sham cells were incubated in high glucose DMEM containing 10% FBS without oxygen deprivation.

### Cell Viability Assay

The CCK-8 assay was performed to detect BV2 microglial cell viability at baseline or following OGD/R. A 10 μl CCK-8 solution was added to each well and incubated for 2 h. The absorbance at 450 nm was detected using a plate reader.

### Immunofluorescence and Double Immunofluorescent Staining

Based on the results of a previous study (Hu et al., 2015), iNOS and Arg1 were considered markers of M1 and M2 microglia activation, respectively. After the OGD/R experiments, the microglial cells were washed with phosphate-buffered saline (PBS) and fixed with ice-cold 4% formaldehyde for 30 min at room temperature, permeabilized with 0.1% Triton X-100, and blocked with normal goat serum at room temperature. Then, the cells were incubated overnight at 4°C with primary antibodies rabbit anti-iNOS (1:50) or mouse anti-Arg1 (1:50), followed by incubation with CoraLite594-conjugated goat anti-rabbit IgG (1:100) or CoraLite488-conjugated goat anti-mouse IgG (1:100), respectively. Equivoluminal secondary antibody was added to another sample stimulated by 30 min to serve as a negative control. The slides were counterstained with 4, 6-diamidino-2-phenylindole dihydrochloride (DAPI) in the dark for 30 min. For double immunofluorescent staining, the cells were incubated overnight with mouse anti-ubiquitin antibody (1:50). After 24 h, the slices were washed and incubated with rabbit anti-VDAC3 (1:100) for 2 h. Then, the cells were washed and incubated with CoraLite-conjugated secondary antibody (1:100) at room temperature for 1 h and the nuclei were stained with DAPI. Five visual fields at a magnification of 200× were randomly captured from each well using an inverted fluorescence microscope (Leica DMi8, Germany); the fluorescence intensities in each visual field were measured using ImageJ software. For the quantification of double immunofluorescent staining, the colocalization coefficient between ubiquitin and VDAC3 displayed as Pearson coefficients in the colocalized volume (1, perfect correlation; 0, no correlation; −1, perfect inverse correlation).

### ELISA for TNF-α, IL-1β, and IL-10

The concentrations of TNF-α, IL-1β, and IL-10 in cell supernatants were determined using commercial ELISA test kits, according to the manufacturer’s instructions. The OD value was read using a microplate reader set to a test wavelength of 450 nm and corrected for absorbance at 540 nm. Quantification was performed using the standard curve.

### Flow Cytometric Analysis

A flow cytometer (BD FACSCalibur, Franklin Lakes, NJ, USA) was used to evaluate the mitochondrial membrane potential (Δψm). After reoxygenation, each group of cells was collected and resuspended in PBS. The working solution of JC-1 staining was mixed well and incubated at 37°C for 15 min according to the manufacturer’s protocol. After washing thrice with the working buffer, the cells were detected using a flow cytometer.

### Western Blot Analysis

Protein concentrations were quantified using the Bradford protein assay kit (Bio-Rad Laboratories). The protein samples (30 μg/lane) were electrophoresed on 15% sodium dodecyl sulfate (SDS)-polyacrylamide gel and electrotransferred to a polyvinylidene fluoride (PVDF) membrane (Bio-Rad Laboratories). The membrane was blocked with 5% skim milk for 1 h at room temperature and incubated overnight at 4°C with the corresponding primary antibodies: caspase3 (1:1,000), VDAC3 (1:1,000), or β-actin (1:2,000). After the membrane was washed in Tris-buffered saline-Tween 20 (TBST), it was incubated with horseradish peroxidase-conjugated goat anti-rabbit or anti-mouse IgG (1:20,000). β-Actin was used as an internal control to normalize the relative expression of caspase3, cleaved caspase3, and VDAC3. The optical densities of the protein bands were analyzed using ImageJ software.

### Immunoprecipitation (IP) and Immunoblotting

To analyze VDAC3 using IP assays, cells harvested from culture dishes were lysed in radioimmunoprecipitation assay (RIPA; Solarbio, China) lysis buffer containing 1 mM phenylmethanesulfonyl fluoride (PMSF; Boster, China), followed by centrifugation (12,000× *g*, 4 °C, 30 min). Then, protein concentrations were measured using bicinchoninic acid (BCA) reaction. According to the instructions, the lysates (1 mg/lane) were incubated with 100 μl protein G magnetic beads (Bio-Rad Laboratories) and 5 μg anti-ubiquitin antibodies at 4°C overnight with agitation. The immunoprecipitated proteins were separated *via* SDS-polyacrylamide gel electrophoresis (PAGE), transferred to PVDF membranes, and probed with ubiquitin antibody (1:1,000), VDAC3 antibodies (1:1,000), followed by incubation with horseradish peroxidase-conjugated secondary antibodies (1:20,000). β-Actin was used as an internal control to normalize the relative expression of ubiquitin-related proteins. The optical densities of the protein bands were analyzed using the ImageJ software.

### RNA Isolation and Quantitative Polymerase Chain Reaction (qPCR)

VDAC3 mRNA was quantified using reverse transcription qPCR using an ABI ViiA7 system (Thermo Fisher Scientific). Total RNA from BV2 cells was isolated using the RNAprep Pure Cell kit according to the manufacturer’s instructions. The concentration of the isolated total RNA was calculated from the absorbance at 260 nm using a spectrophotometer (Bio-Rad), and the purity was verified using the optical density absorption ratio (260 nm/280 nm), which was between 1.8 and 2.0. Reverse transcription was performed using the first-strand cDNA synthesis kit with random primers to synthesize single-stranded cDNA. The reaction conditions were as follows: 25°C for 10 min, then 42°C for another 15 min, followed by 85°C for 5 min. All cDNA samples for the subsequent qPCR analyses were stored at −20°C. Then, qPCR was performed using the EvaGreen 2× qPCR master mix kit at 95°C for 10 min, then denaturation at 95°C for 15 s, followed by 60°C for 60 s. The relative amount of the target gene’s mRNA was calculated using the 2^−ΔΔCt^ method. Each sample was normalized based on the β-actin expression. The melting curves for each PCR reaction were analyzed to ensure the purity of the amplification product. The sequences of the primers were as follows, VDAC3: 5′- TGGTTCGAGAAGACCTTCAGC-3′ (forward), and 5′-AGGTCGCAGTAAGTTGGTGT-3′ (reverse); β-actin: 5′- ACTCATCGTACTCCTGCTTG-3′ (forward), 5′-GAAATCGTGCGTGACATTA-3′ (reverse). The primers were designed using NCBI.

### Statistical Analysis

The data were expressed as mean ± standard deviation of the mean ($\bar{x}$ ± SD). The SPSS 22.0 software (IBM, New York, NY, USA) was used for statistical analysis. After confirmation of normal distribution with the Kolmogorov-Smirnov-Test, all variables were compared using a one-way analysis of variance (ANOVA), and pairwise comparison between sample means was performed using the Bonferroni method. Comparisons between baseline and OGD or OGD/R were evaluated using a paired Student’s *t*-test. *P* < 0.05 indicated statistically significant differences.

## Results

### Detection of Cell Viability Using the CCK-8 Assay

**Figure 1:**
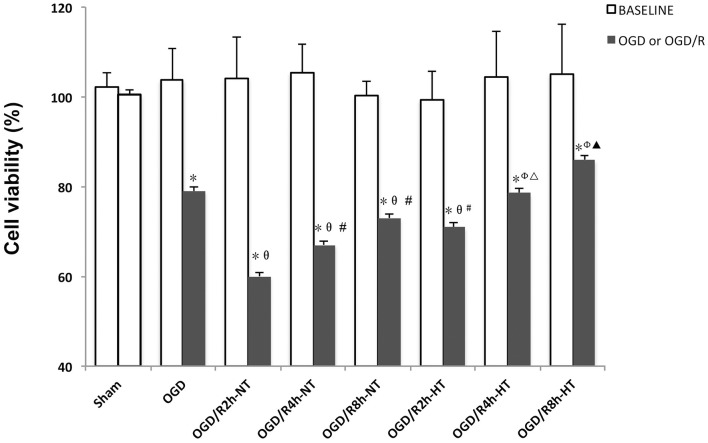
Effects of hypothermia on oxygen-glucose deprivation, followed by recovery (OGD/R)-induced cytotoxicity in microglia. Cell viability was determined using the cell counting kit-8 (CCK-8) assay. Values show the mean ± SD (*n* = 5). *P < 0.05, vs. Sham group; ^θ^*P* < 0.05, vs. OGD group; ^φ^*P* < 0.05, vs. OGD/R2h-HT groups; ^#^*P* < 0.05, vs. OGD/R2h-NT group; ^▵^*P* < 0.05, vs. OGD/R4h-NT group; ^▴^*P* < 0.05, vs. OGD/R8h-NT group. White column = baseline; black solid column = OGD or OGD/R groups.

The BV2 cell viability before and following treatment was measured using the CCK-8 assay. As shown in Figure 1, there were no significant differences in OD values at baseline between the groups. Compared to the baseline, the OD values were significantly reduced in both OGD and OGD/R groups (*P* < 0.05). Cell viability was further reduced following OGD, reached to the minimum at OGR 2 h, and then gradually increased (*P* < 0.05). However, significantly higher viability was observed in the HT groups than that in the NT groups at OGR 2, 4, and 8 h, respectively (*P* < 0.05).

### Hypothermia Regulated OGD/R-Induced BV2 Microglial Activation

**Figure 2:**
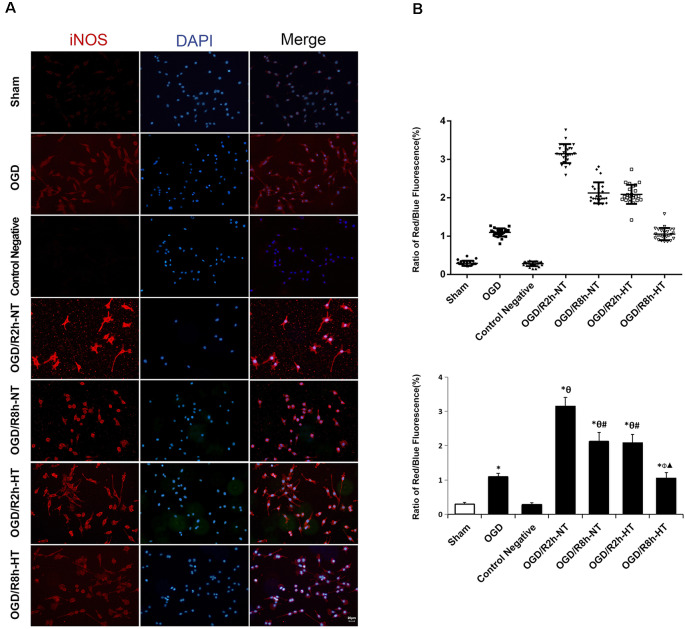
Effects of hypothermia on the immunoreactivity of anti-induced nitric oxide synthase (iNOS)-positive microglia using immunofluorescence assays.** (A)** Immunofluorescence images showing the BV2 microglia following OGD/R labeled with the iNOS antibody or secondary antibody (control negative). Red fluorescence indicates iNOS-positive cells, while blue fluorescence indicates 4,6-diamidino-2-phenylindole dihydrochloride (DAPI)-labeled nuclei. Scale bar: 20 μm.** (B)** Dot plots (upper panel) and a bar graph (lower panel) showing a quantitative analysis of red/blue fluorescence ratios (25 random observations for each group). Values show the mean ± SD, *n* = 5. *P < 0.05, vs. sham group; ^θ^*P* < 0.05, vs. OGD group; ^φ^*P* < 0.05, vs. OGD/R2h-HT groups; ^#^*P* < 0.05, vs. OGD/R2h-NT groups; ^▴^*P* < 0.05, vs. OGD/R8h-NT groups.

**Figure 3:**
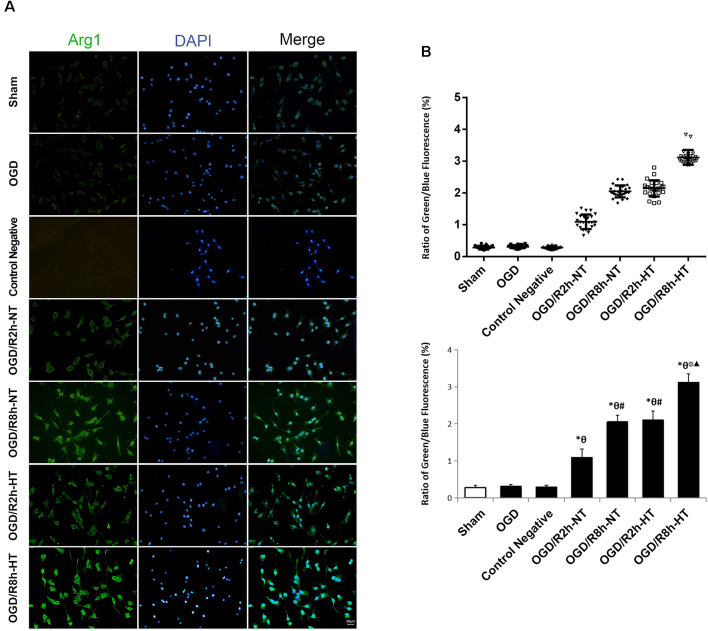
Effects of hypothermia on the immunoreactivity of arginase 1 (Arg1)-positive microglia using immunofluorescence assays. **(A)** Immunofluorescence images showing the BV2 microglia following OGD/R labeled with the Arg1 antibody or secondary antibody (control negative). Green fluorescence indicates Arg1-positive cells, while blue fluorescence indicates DAPI-labeled nuclei. Scale bar: 20 μm.** (B)** Dot plots (upper panel) and a bar graph (lower panel) showing a quantitative analysis of green/blue fluorescence ratios (25 random observations for each group). Values show the mean ± SD, *n* = 5. *P < 0.05, vs. sham group; ^θ^*P* < 0.05, vs. OGD group; ^φ^*P* < 0.05, vs. OGD/R2h-HT groups; ^#^*P* < 0.05, vs. OGD/R2h-NT groups; ^▴^*P* < 0.05, vs. OGD/R8h-NT groups.

Cellular immunofluorescence showed that the BV2 microglia cells responded to OGD/R acquired amoeboid morphology, characterized by retraction and thickening of the processes, and hypertrophy of the cell body. Concurrently, microglial activation increased as observed by an early increase in iNOS. The fluorescence intensity of iNOS following OGD continued to increase and peaked at OGR 2 h and then decreased OGR 8 h (*P* < 0.05). However, hypothermia reduced iNOS-positive immunostaining, with the morphological transformation of the microglia, in comparison to that observed in normothermia-treated samples at 2 h and 8 h following reperfusion (*P* < 0.05). Compared to the sham group, the Arg1 level increased gradually with progress in reperfusion and peaked at OGR 8 h (*P* < 0.05). However, hypothermia significantly increased the expression of Arg1 at 2 h and 8 h, respectively (*P* < 0.05). Taken together, these results indicated that hypothermia might promote the shift of M1 to M2 microglia (Figures 2, 3).

### Hypothermia Refined OGD/R-Induced Release of Inflammatory Cytokines in the Microglia

**Figure 4:**
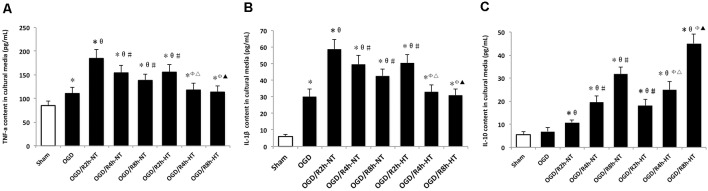
Effects of hypothermia on pro-inflammatory and anti-inflammatory cytokine release in OGD/R-induced microglia. Protein levels of interleukin (IL)-1β **(A)** tumor necrosis factor (TNF)-α **(B)** and IL-10 **(C)** in culture media were detected using enzyme-linked immunosorbent assay (ELISA). Values show the mean ± SD, *n* = 5. *P < 0.05, vs. sham group; ^θ^*P* < 0.05, vs. OGD group; ^φ^*P* < 0.05, vs. OGD/R2h-HT groups; ^#^*P* < 0.05, vs. OGD/R2h-NT groups; ^▵^*P* < 0.05, vs. OGD/R4h-NT groups; ^▴^*P* < 0.05, vs. OGD/R8h-NT groups.

Pro-inflammatory and anti-inflammatory responses were induced by OGD/R in cultured BV2 cells. Compared to the sham group, the protein levels of TNF-α, IL-1β, and IL-10 in the culture media were increased in both OGD and OGD/R groups. Pro-inflammatory cytokines (TNF-α and IL-1β) were further increased following OGD, peaked at OGR 2 h, and then gradually decreased during reperfusion (*P* < 0.05). However, TNF-α and IL-1β were markedly lower in the hypothermia groups than that in the normothermia groups at 2, 4, and 8 h following reperfusion (*P* < 0.05). Furthermore, reperfusion increased the level of anti-inflammatory cytokines (IL-10) when compared with the OGD group. However, compared to the normothermic treatment, hypothermia increased the level of IL-10 at both OGR 4 h and OGR 8 h (*P* < 0.05; Figure 4).

### Hypothermia Inhibited OGD/R-Induced Apoptosis in Microglia

**Figure 5:**
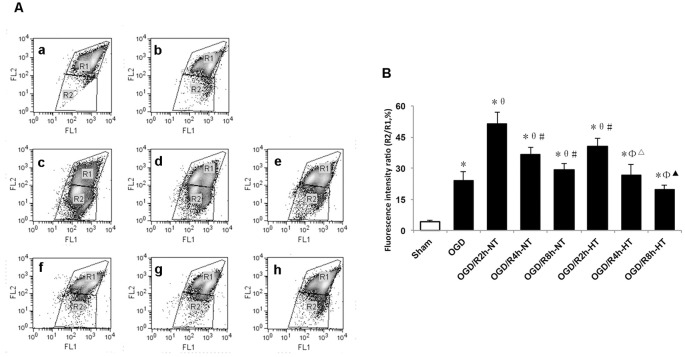
Effect of hypothermia on the loss of mitochondrial membrane potential (Δψm) in microglial cells. **(A)** Scatter diagram of5,5’, 6,6’-tetrachloro-1,1’, 3,3’-tetraethyl-benzimidazolylcarbocyanine iodide (JC-1) dye from different groups. a. Sham; b. OGD; c. OGD/R2h-NT; d. OGD/R4h-NT; e. OGD/R8h-NT; f. OGD/R2h-HT; g. OGD/R4h-HT; h. OGD/R8h-HT. **(B)** Bar graph showing the fluorescence ratio R2/R1 in the different groups. Values show the mean ± SD (*n* = 5). *P < 0.05, vs. Sham group; ^θ^*P* < 0.05, vs. OGD group; ^φ^*P* < 0.05, vs. OGD/R2h-HT groups; ^#^*P* < 0.05, vs. OGD/R2h-NT groups; ^▵^*P* < 0.05, vs. OGD/R4h-NT groups; ^▴^*P* < 0.05, vs. OGD/R8h-NT groups.

To evaluate the role of hypothermia in I/R-induced microglial cytotoxicity, Δψm was assayed using JC-1 staining and a flow cytometer. In healthy mitochondria, JC-1 displayed red fluorescence in the R1 region, whereas in the damaged mitochondria, JC-1 displayed green fluorescence in the R2 region. Δψ_m_ was quantified by the green to red fluorescence ratio, and an increase in the ratio indicated initiation of apoptosis. Compared to the sham group, the ratio was significantly higher in both the OGD and OGD/R groups (*P* < 0.05). The ratio was further increased following OGD, peaked at OGR 2 h, and then gradually decreased (*P* < 0.05). However, the ratio in the HT groups was significantly lower than that in the NT groups at OGR 2, 4, and 8 h, respectively (*P* < 0.05; Figure 5).

### Hypothermia Down-Regulated the Expression of Apoptosis-Associated Proteins in OGD/R-Induced Microglia

**Figure 6:**
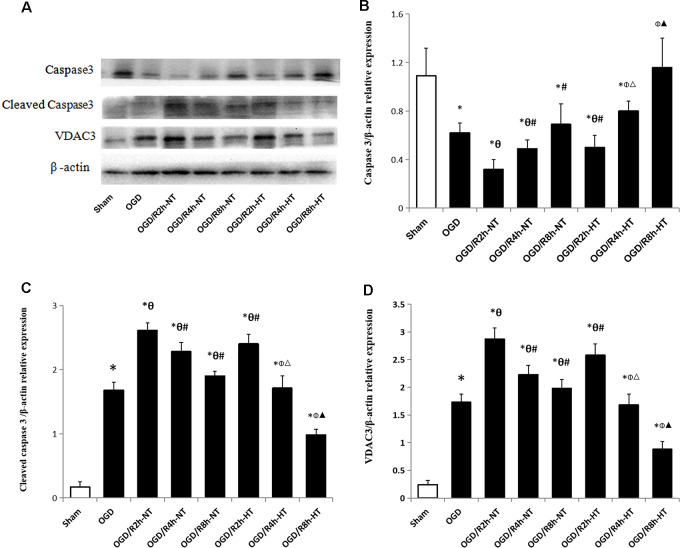
Effect of hypothermia on the expression of apoptosis-associated proteins in microglial cells.** (A)** Representative western blot images showing the expression of caspase3, cleaved caspase3, and voltage-dependent anion channel 3 (VDAC3) in different groups. β-Actin was used as an internal control. **(B)** Bar graph showing the quantification of capase3/β-actin in different groups. **(C)** Bar graph showing the quantification of cleaved capase3/β-actin in different groups. **(D)** Bar graph showing the quantification of cleaved VDAC3/β-actin in different groups. Values show the mean ± SD (*n* = 5). *P < 0.05, vs. Sham group; ^θ^*P* < 0.05, vs. OGD group; ^φ^*P* < 0.05, vs. OGD/R2h-HT groups; ^#^*P* < 0.05, vs. OGD/R2h-NT groups; ^▵^*P* < 0.05, vs. OGD/R4h-NT groups; ^▴^*P* < 0.05, vs. OGD/R8h-NT groups.

To further analyze the apoptosis triggered by simulated I/R, the expression of caspase-3 and cleaved caspase-3, as well as VDAC3, was analyzed using western blot analysis. Compared to the sham group, the expression of caspase-3 was significantly reduced in both the OGD and OGD/R groups (*P* < 0.05). Reperfusion at 2 h further decreased the expression of caspase-3 when compared with the OGD group (*P* < 0.05). However, the expression of caspase-3 in the NT groups was lower than that in the HT groups at OGR 2, 4, and 8 h (*P* < 0.05). Instead, both the OGD and OGD/R groups demonstrated significantly higher levels of cleaved caspase-3 and VDAC3 than the sham group (*P* < 0.05). Both cleaved caspase-3 and VDAC3 were further increased following OGD, peaked at OGR 2 h, and then gradually decreased during reperfusion (*P* < 0.05). However, the expression of cleaved caspase-3 and VDAC3 in the HT group was significantly lower than that in the NT groups at OGR 2, 4, and 8 h, respectively (*P* < 0.05). These results suggested that hypothermia-mediated attenuation of OGD/R-induced apoptosis might be associated with a reduced level of VDAC3 (Figure 6).

### Hypothermia Up-Regulated Ubiquitinated VDAC3 Protein Level in OGD/R-Induced Microglia

**Figure 7:**
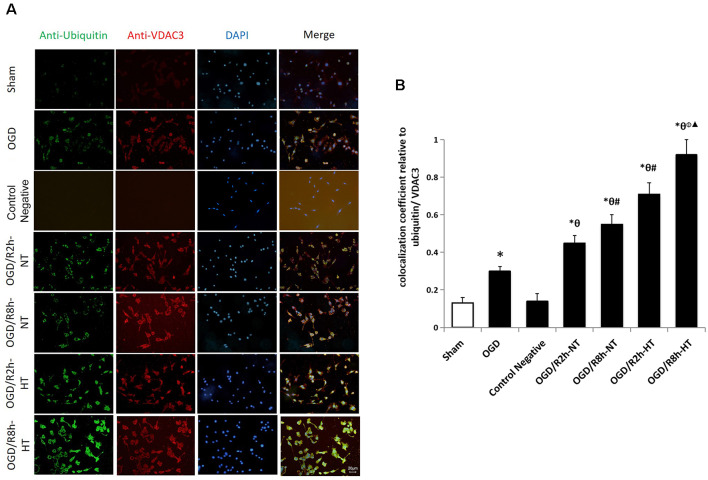
Effects of hypothermia on the immunoreactivity of ubiquitin-positive and anti-VDAC3-positive microglia using double immunofluorescence assays.** (A)** Immunofluorescence images showing the BV2 microglia following OGD/R labeled with ubiquitin (green) and VDAC3 (red) antibody or secondary antibodies (control negative). Blue fluorescence indicates DAPI-labeled nuclei. Scale bar: 20 μm.** (B)** Quantification of the colocalization coefficient between ubiquitin and VDAC3. Values show the mean ± SD, *n* = 5. *P < 0.05, vs. sham group; ^θ^*P* < 0.05, vs. OGD group; ^φ^*P* < 0.05, vs. OGD/R2h-HT groups; ^#^*P* < 0.05, vs. OGD/R2h-NT groups; ^▴^*P* < 0.05, vs. OGD/R8h-NT groups.

Protein ubiquitination was further used to detect the cause of hypothermia-induced down-regulation of VDAC3. Double immunofluorescent staining using anti-ubiquitin (green) and anti-VDAC3 (red) identified the cellular location of ubiquitin-related proteins and VDAC3. As indicated in Figure 7, the colocalization of ubiquitin and VDAC3 was significantly increased after OGD and peaked at OGR 8 h in both NT groups and HT groups (*P* < 0.05). However, the co-expression of ubiquitin and VADC3 in the HT groups was significantly higher than that in the NT groups at OGR 2 h and 8 h, respectively (*P* < 0.05).

**Figure 8:**
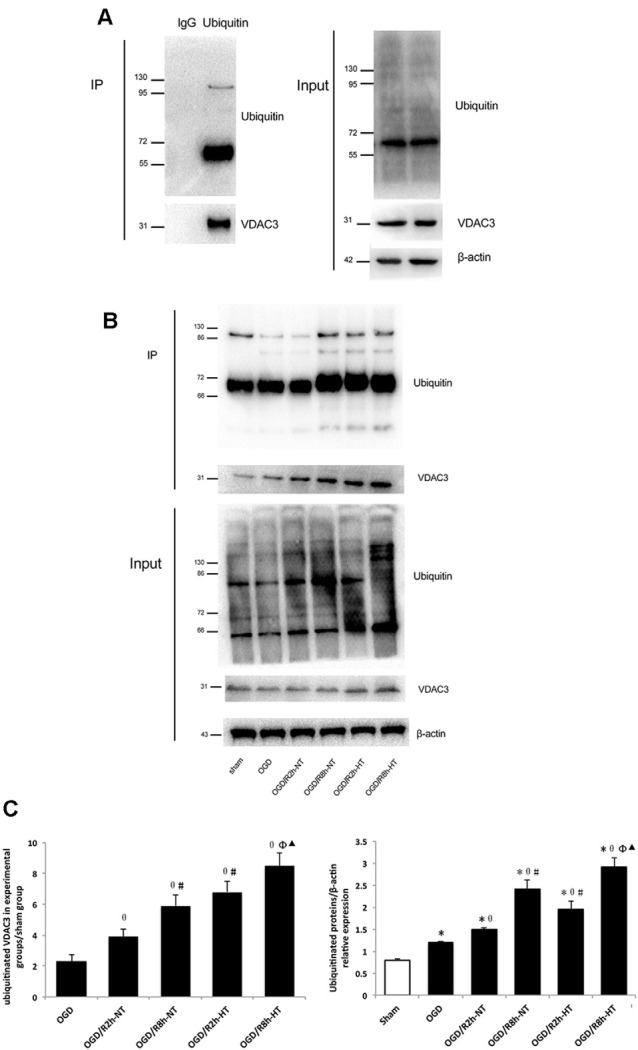
Hypothermia induced ubiquitination of VDAC3 in microglial cells. **(A)** To detect non-specific binding with magnetic beads, OGD/R8h-HT cells were immunoprecipitated with IgG or anti-ubiquitin, followed by western blotting (WB) with anti-VDAC3 and anti-ubiquitin antibodies. Anti-ubiquitin and anti-VDAC3 were used for WB to determine the ubiquitination level of VDAC3 (left). WB data of ubiquitin, VDAC3, and β-actin from the input (right). **(B)** Immunoblot analyses of ubiquitinated VDAC3 in different groups. Cell lysates were immunoprecipitated with anti-ubiquitin antibody, followed by WB with the anti-ubiquitin antibody and VDAC3 antibodies. β-Actin was used as an internal control of input. **(C)** Quantification of ubiquitinated VDAC3 in experimental groups/sham group (Left) and ubiquitinated proteins/β-actin in different groups (right). Results are shown as mean ± SD (*n* = 5). Left: *P < 0.05, vs. Sham group; ^θ^*P* < 0.05, vs. OGD group; ^φ^*P* < 0.05, vs. OGD/R2h-HT groups; ^#^*P* < 0.05, vs. OGD/R2h-NT groups; ^▴^*P* < 0.05, vs. OGD/R8h-NT groups.

As shown in Figure 8A, the binding of VDAC3 to the ubiquitin was first verified. Indeed, the levels of ubiquitin-related proteins were significantly higher in both OGD and OGD/R groups than in the sham group (*P* < 0.05; Figure 8B input, 8C-Right). As expected, reperfusion at 8 h further increased ubiquitinated VDAC3 when compared with OGR 2 h (*P* < 0.05; Figure 8B IP, Figure 8C-Left). However, the expression of ubiquitinated VDAC3 in the HT group was considerably higher than that in the NT group at OGD/R 2 h and 8 h, respectively (*P* < 0.05; Figure 8B IP, Figure 8C-Left). These data suggest that hypothermia promotes the degradation of VDAC3 *via* the ubiquitination pathway.

### Hypothermia Did Not Attenuate VDAC3 mRNA Expression in OGD/R-Induced Microglia

**Figure 9:**
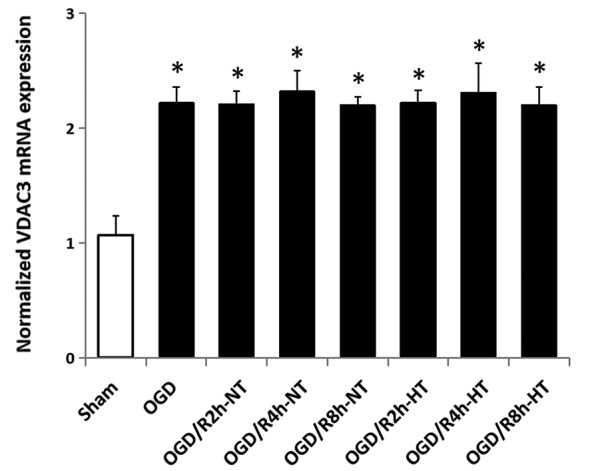
Effect of hypothermia on VDAC3 mRNA expression in microglial cells. The normalized VDAC3 mRNA level was determined in each group using real-time RT-PCR. *P < 0.05, vs. sham group. *n* = 5 repeated from five independent experiments.

The VDAC3 mRNA level was significantly higher in both OGD and OGD/R groups than in the sham group (*P* < 0.05). However, there was no significant difference in VDAC3 mRNA expression between the NT group and HT group at 2, 4, or 8 h following the reperfusion (*P* > 0.05; Figure 9).

## Discussion

The present *in vitro* study demonstrated that OGD/R induced BV2 microglial activation, inflammation, and apoptosis, and the cytotoxicity was reduced after exposure to hypothermia. Furthermore, we observed that VDAC3 was significantly up-regulated following OGD/R injury and probably participated in apoptosis. Accordingly, hypothermia exerted a neuroprotective effect against OGD/R, probably by facilitating ubiquitination and VDAC3 degradation. Also, the longer the hypothermia duration, the greater ubiquitination of VDAC3 was observed. Thus, therapeutic hypothermia targeting microglia may have clinical significance for patients with cardiac arrest and acute cerebral ischemia/reperfusion.

Increasing evidence shows that different microglia phenotypes perform diverse physiopathological functions after brain ischemia, trauma, or infection (Wang et al., 2013; Ma et al., 2017; Luo et al., 2018). In this study, we observed that microglia activation occurred in a time-dependent manner following cerebral I/R injury. The inflammatory phenotype, known as the M1 phenotype, was characterized by the production of pro-inflammatory cytokines (TNF-α, IL-1β), and presented iNOS immunostaining at the early stage of oxygen-glucose reperfusion. In acute ischemic stroke, M1 microglia was associated with inflammation and apoptosis, leading to tissue injury or neurological deficits (Cheon et al., 2017; McCrary et al., 2019). However, the anti-inflammatory or M2 phenotype was capable of producing anti-inflammatory cytokines (IL-10) as well as higher levels of Arg1 at the late stage of reperfusion. Activated microglia could also function in a neuroprotective manner and contribute to tissue repair and regeneration by removing debris and secreting anti-inflammation or glial cell-derived neurotrophic factor during the extended period (Zhao et al., 2019). These findings were partially consistent with the radiological or molecular data that dynamically evaluated microglia activation *in vivo* (Gerhard et al., 2005; Price et al., 2006; Thiel and Heiss, 2011). Our results also suggested that hypothermia inhibited early inflammation and apoptosis, or induced microglia transformation, which might be a therapeutic strategy for cerebral I/R.

Convincing evidence supports the notion that a major increase in the levels of ubiquitination or ubiquitin-like proteins induces tolerance to ischemic stress (Meller, 2009; Lee and Hallenbeck, 2013; Bernstock et al., 2018). In the present study, hypothermia was sufficient to stimulate ubiquitin-related proteins against OGD/R injury in the BV2 microglia. This result was as per those of previous studies. In rats subjected to cardiopulmonary bypass at 18°C, the levels of small ubiquitin-like modifier (SUMO)-conjugated proteins were high in all organs tested (Yang et al., 2009). Moderate hypothermia-induced SUMO conjugation also reduced damage to bone marrow stromal cells (BMSCs) following OGD and improved BMSC survival following transplantation into the penumbra (Liu et al., 2017; Ren et al., 2017). Similarly, hibernation-like hypothermia induced by opioid receptor agonists protected the brain from ischemic injury (Lee et al., 2007; Crowley et al., 2017). Therefore, it will be interesting to understand the connection between hibernation, ubiquitination, and neuroprotection. Accordingly, drugs that activate ubiquitination or opioid receptors may provide a suitable strategy for clinical use. Thus, the present study indicated that hypothermia promoted ubiquitin-related proteins and induced VDAC3 degradation, which might be a probable mechanism underlying the neuroprotection of hypothermia in resistance to OGD/R.

As an essential component and regulator of mitochondrial permeability transition pore (mPTP), VDAC contributes to loss of mitochondrial membrane potential, mitochondrial swelling, and rupture of the OMM during I/R injury. Several studies have directly measured VDAC activity (Lai et al., 2006; Roman et al., 2006); however, purified VDAC in a planar bilayer might respond differently than VDAC *in situ*. Immunohistochemical and western blot analyses showed overexpression of VDAC1 following cerebral ischemia/reperfusion injury in rats (Li et al., 2018). Instead, the inhibition of VDAC is involved in the tolerance of liver graft to I/R injury associated with liver transplantation (Zaouali et al., 2017). Furthermore, mitochondrial [Ca^2+^] fluctuation is attributed to VDAC overexpression, whereas ischemic post-conditioning protected against mitochondrial damage by stabilizing VDAC (Yao et al., 2018). These data indicated that both transcriptional regulation and post-translational modifications of VDAC were associated with mitochondrial function. This is the first study to report that overexpression of VDAC3 is associated with microglial I/R injury, while hypothermia facilitated microglial protection *via* ubiquitination and degradation of VDAC3.

It should be noted that the current study has several limitations. First, the amino acid sequence of VDAC3 is conserved across species; however, further studies on the human microglia or *in vivo* experiments are required to identify the different functions of VDAC3. Second, VDAC was originally characterized as a mitochondrial porin; however, evidence shows that VDAC was also expressed in the plasma membranes of human cells (De Pinto et al., 2010). Thus, we could not specify whether functional VDAC3 expressed from the mitochondria or the plasma membrane. Finally, the results of IP suggested that ubiquitination is required for targeting proteins; therefore, protein mass spectrometry analysis is required to exclude the possibility that VDAC activity is modulated by protein interactions and other post-translational modifications.

In summary, the present study suggested that hypothermia protected BV2 microglia from I/R-induced inflammation and apoptosis. This may be due to the enhancement of ubiquitination and VDAC3 degradation. Thus, the endogenous protective pathway of ubiquitination may be a potential target for developing novel therapeutic strategies in the future.

## Data Availability Statement

The raw data supporting the conclusions of this article will be made available by the authors, without undue reservation, to any qualified researcher.

## Author Contributions

ZT, SZ, and FC contributed to the conception and design of the study. PX, HC, and CL organized the database. HC and PG performed the statistical analysis. SZ wrote the first draft of the manuscript. All authors contributed to manuscript revision, read and approved the submitted version.

## Conflict of Interest

The authors declare that the research was conducted in the absence of any commercial or financial relationships that could be construed as a potential conflict of interest.
